# Tumor glucose metabolism and the T cell glycocalyx: implication for T cell function

**DOI:** 10.3389/fimmu.2024.1409238

**Published:** 2024-05-31

**Authors:** Fabian Schuurmans, Kyra E. Wagemans, Gosse J. Adema, Lenneke A. M. Cornelissen

**Affiliations:** Radiotherapy and OncoImmunology Laboratory, Department of Radiation Oncology, Radboud University Medical Center, Nijmegen, Netherlands

**Keywords:** tumor microenvironment, metabolism, T cell glycocalyx, glycobiology, tumor immunity

## Abstract

The T cell is an immune cell subset highly effective in eliminating cancer cells. Cancer immunotherapy empowers T cells and occupies a solid position in cancer treatment. The response rate, however, remains relatively low (<30%). The efficacy of immunotherapy is highly dependent on T cell infiltration into the tumor microenvironment (TME) and the ability of these infiltrated T cells to sustain their function within the TME. A better understanding of the inhibitory impact of the TME on T cells is crucial to improve cancer immunotherapy. Tumor cells are well described for their switch into aerobic glycolysis (Warburg effect), resulting in high glucose consumption and a metabolically distinct TME. Conversely, glycosylation, a predominant posttranslational modification of proteins, also relies on glucose molecules. Proper glycosylation of T cell receptors influences the immunological synapse between T cells and tumor cells, thereby affecting T cell effector functions including their cytolytic and cytostatic activities. This review delves into the complex interplay between tumor glucose metabolism and the glycocalyx of T cells, shedding light on how the TME can induce alterations in the T cell glycocalyx, which can subsequently influence the T cell’s ability to target and eliminate tumor cells.

## 1 Immunotherapy to reinvigorate T cell effector functions

The T cell is an immune cell subset highly effective in eliminating cancer cells. Upon priming by professional antigen presenting cells that present tumor (neo-)antigens, T cells become activated and can recognize cancer cells. T cell activation induces rapid T cell proliferation leading to the expansion of a population of T cells specifically targeting the cancer cell (1). The resulting effector T cells can directly kill cancer cells by releasing cytotoxic molecules such as perforin and granzymes, which induce programmed cell death through a multi-hit mechanism (2). Additionally, T cells can induce apoptosis in cancer cells through interactions involving death receptors and ligands, such as Fas ligands (FasL) binding to Fas receptor on the surface of cancer cells (3). Furthermore, T cells can release cytokines such as interferon-gamma (IFNy) and tumor necrosis factor-alpha (TNFα), which have anti-tumor effects by inducing permanent growth arrest, leading to their elimination and promotion of inflammation (4).

Cancer cells evolve, however, mechanisms to evade T cell-mediated killing. Immune checkpoints, both stimulatory and inhibitory receptors, control and co-determine the functional outcome of T cell effector responses (5, 6). Immune checkpoint receptor/ligand pairs are present on a diverse set of cells where they regulate the initiation and course of the immune response, which otherwise can cause tissue damage or the development of autoimmunity. Immune checkpoint therapy aims to release the break and harness the body’s immune system to enhance its ability to recognize and destroy cancer cells. The most targeted immune checkpoints in the onco-immunology field are PD-1, PD-L1 and CTLA-4. Monoclonal antibodies blocking these checkpoints interfere with T cell feedback loops and empowers T cells to eliminate cancer cells. Immune checkpoint therapy has shown significant clinical benefit for subgroups of patients with different malignancies (7–11), however, the overall response rate remains below 30% (12).

Next to immune checkpoint therapy, CAR T cell therapy constitutes another form of immunotherapy within the field of oncology. CAR T cell therapy involves genetically modifying a patient’s T cell to express chimeric antigen receptors (CARs) that target specific antigens on cancer cells including glycosylated antigens (13–15). Such ex-vivo engineered CAR T cells are then infused back into the patient, where they can target and kill cancer cells expressing the corresponding antigen. CAR T cells therapy has shown remarkable success especially in hematologic malignancies (16). Despite is advancements, there are still many challenges and questions to address regarding CAR T cell therapies. Factors such as CAR T cell exhaustions and antigen escape contribute to treatment resistance and the occurrence of adverse events highlight the importance of monitoring and managing treatment-related complications (17). As an example, hyperglycosylation of the CAR T cell antigen CD19 directly inhibits CAR T cell effector functions, leading to less T cell cytotoxicity (18). In solid tumors, CAR T cell therapy has shown limited clinical efficacy due to factors such as inadequate tumor infiltration and an immunosuppressive tumor microenvironment (19).

T cell cytotoxicity requires multi-hit delivery to induce cell death and in the absence of suppressive signaling, T cells are capable to engage and eliminate multiple cancer cells successively (serial killing). Hence the efficacy of cancer immunotherapy is not solely dictated by T cell infiltration but also by the ability of the (CAR) T cells to sustain their functions within the TME. Obtaining more insights into the inhibitory impact of the TME on T cells is indispensable to improve immunotherapy. A century ago, Otto Heinrich Warburg noted a distinct contrast between the TME and non-malignant tissues in terms of metabolism. Tumor cells exhibit altered glucose metabolism, leading to high glucose consumption, which results in lactate production and acidification of the TME, commonly known as the Warburg effect (20, 21).

## 2 Tumor metabolism

**Figure 1:**
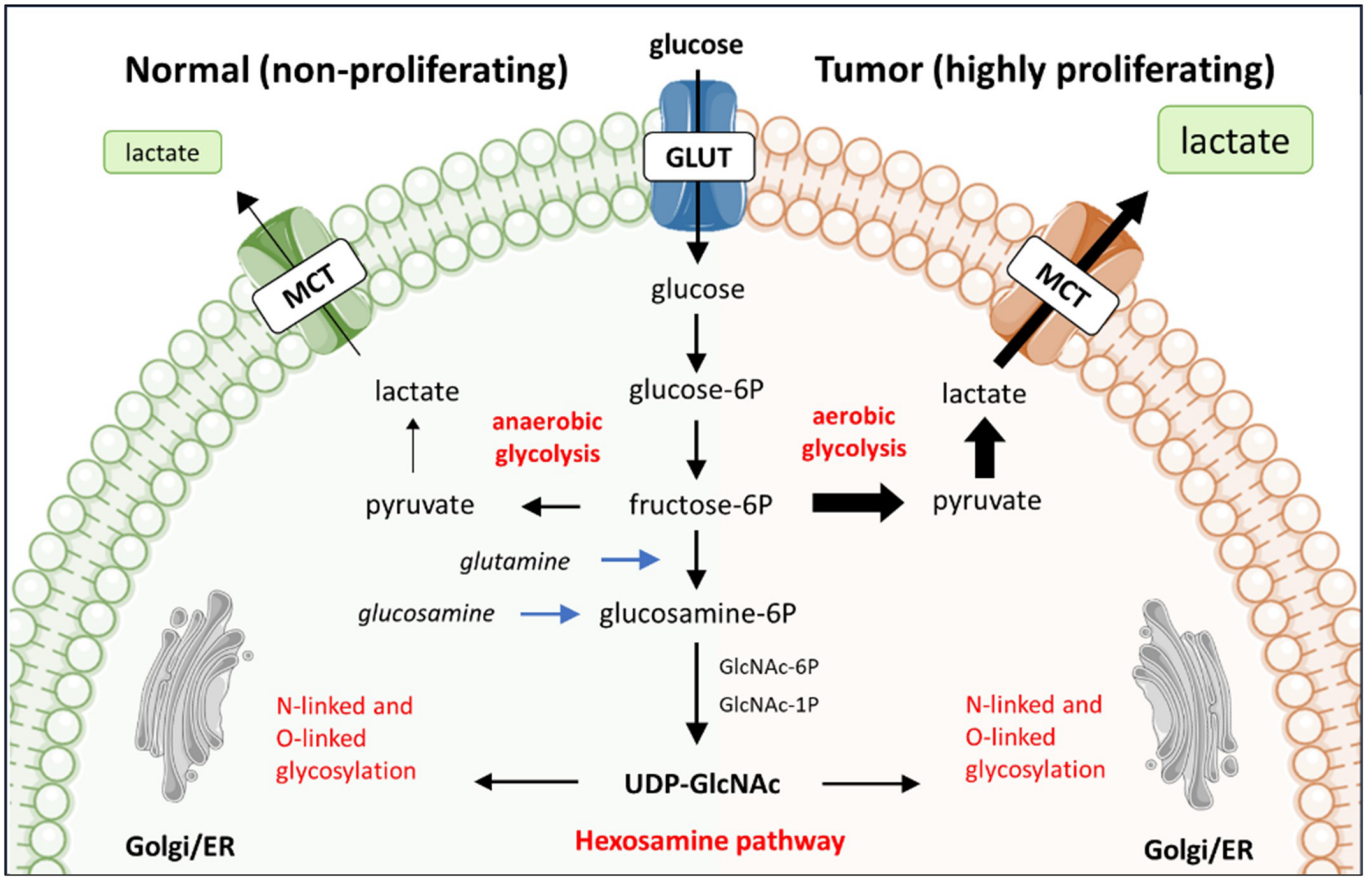
The preferential use of aerobic glycolysis by tumor cells and its relationship with glycosylation. The reliance of tumor cells on aerobic glycolysis, rather than oxidative phosphorylation, leads to heightened lactate production from glucose metabolism. This preference for aerobic glycolysis is associated with the hexosamine biosynthesis pathway (HBP), which shares the initial steps of glucose metabolism. The HBP is pivotal in protein and lipid glycosylation, thereby establish a significant interconnection between glycosylation and glucose metabolism.

Tumor cells exhibit a heightened need for energy to sustain their uncontrolled proliferation and to ensure survival. The Warburg effect involves preferential use of glucose for aerobic glycolysis even in the presence of oxygen. In non-cancerous context, the Warburg effect is exploited by rapidly proliferating cells. In tumor cells, the Warburg effect is a well-known metabolic alteration to support their high proliferation rate (**Figure 1**). This metabolic adaptation provides the tumor cells with energy, albeit less efficiently in terms of ATP production than oxidative phosphorylation (20). Tumor cells do favor aerobic glycolysis as it allows for quick energy production, and it provides intermediates that can be used for the biosynthesis of nucleotides, amino acids and lipids, the building blocks for the synthesis of cellular components. Moreover, the increased demand for Nicotinamide adenine dinucleotide (NAD^+^) relative to ATP has been found to drive aerobic glycolysis (22). NAD^+^ and its reduced forms (NADH, NADP^+^ and NADPH) are essential redox metabolites in numerous metabolic processes, acting as hybrid-accepting and donating co-enzymes. The heightened glucose consumption by tumor cells results in a competition for glucose between immune cells and tumor cells and metabolically restricts infiltrating T cells. Together facilitating tumor growth and progression (23). Moreover, aerobic glycolysis culminates in the production of lactate, leading to heightened lactate concentration in the TME when glycolysis is increased. Lactate has been identified as an alternative energy source for tumor cells, as exogenous lactate can serve as a substrate for the tricarboxylic acid (TCA) cycle to fuel cancer cells growth (24, 25). At the same time, lactate causes extracellular acidification, which can facilitate tumor progression by suppressing immune response and promoting tumor invasion (26).

In addition to glucose, tumor cells can utilize glutamine as an alternative energy source. Similarly, the altered glutamine metabolism leads to a reduction in the availability of this essential nutrient for immune cells. The competition for glutamine can impair the function of immune cells, impacting their ability to mount an effective anti-tumor immune response (27). Moreover, tumor cells can switch to lipid metabolism as an alternative source of energy and building materials. Lipid metabolic reprogramming not only supports tumor development, it also modifies the TME by affecting the recruitment, function and survival of infiltrating immune cells (28). Fatty acids are involved in membrane proliferation (29) and can be secreted by the tumor cell to influence the functioning of immune cells (30).

Collectively, the TME displays metabolic abnormalities in comparison to healthy tissue. Research has demonstrated that competition for energy and nutrients hampers immunity. In addition, tumor metabolites affect the efficacy of immune cells, exerting a direct immunosuppressive effect on immune cells (31). The scope of this review is on tumor glucose metabolism and its impact on local T cell glycosylation and function.

## 3 The glucose metabolism pathway and glycosylation biosynthesis are intertwined

Tumor cells utilize high amounts of glucose and glutamine via aerobic glycolysis. These glucose and glutamine molecules, however, are also consumed by the metabolic hexosamine biosynthesis pathway (HBP) in the cytoplasm of the cell. The HBP converts glucose or glutamine to UPD-*N-*acetylglucosamine *(*UPD-GlcNAc) via a six-step pathway that shares the first two steps with glycolysis (**Figure 1**). UDP-GlcNAc is one of the essential intermediates for glycosylation, hence HBP plays a significant role in regulating glycosylation. For instance, low levels of glucose have been observed to reduce the availability of glycosylation precursors in expression systems such as CHO cells. Consequently leading to more non-glycosylated proteins produced by these CHO cells (32). In contrast, supplementation of glucose to primary murine T cell cultures changed the glycosylation profile of the T cells with functional consequences. Specifically, the attachment of β1,6-GlcNAc-branched *N*-glycans to cell surface glycoproteins negatively regulated T cell receptor clustering and signaling at the immune synapse (33), an essential interface between cells needed for proper activation of naïve T cell as well as the ability of effector T cells to kill tumor cells.

Glycosylation is the process of covalently attaching monosaccharides to other monosaccharides, proteins, and lipids, creating a wide repertoire of cellular glycans, collectively referred to as the glycome. There are ten monosaccharide building blocks, which can be modified via phosphorylation, sulfation or acetylation. Unlike DNA/RNA transcription, glycosylation is a non-template driven process and regulated via a wide variety of enzymes (34). The product of HBP, UDP-GlcNAc, serves as a substrate of *O*-GlcNAc transferases (OGT). OGT catalyzes the attachment of a GlcNAc through an *O*-glycosidic linkage to a serine (Ser) or threonine (Thr) residues on intracellular proteins. A delicate on/off competition mechanism between O-GlcNAcylation and phosphorylation takes place on either the same or adjacent Ser/Thr residues. This on/off mechanism regulates the interactions, stability, subcellular localization, and enzymatic activity of shared target proteins involved in essential biological processes (35). Unlike extracellular glycosylation, *O*-GlcNAc is not elongated with monosaccharides to generate more complex glycan structures. In addition to *O*-GlcNAcylation, UDP-GlcNAc serves as a crucial precursor for the biosynthesis of monosaccharides such as UDP-GalNAc and CMP-Neu5Ac that are often utilized in *N*-linked and *O*-linked glycosylation processes (36). Once located at the cell membrane, extracellular glycosylation can be further modified by soluble glycan modifying enzymes including glycosidases and sulfatases (37). It has been described that extrinsic sialyltransferases and glycan substrates, supplied among others by platelets, can modify glycan structures present on cell membranes (38–40). Moreover, monocyte differentiation results in up-regulation of neuraminidase 1 (Neu1) that activates phagocytoses in macrophages and dendritic cells via desialylation of surface receptors (41, 42).

As glycans coat the surface of cells, this posttranslational modification is important in the development of all living organisms (43). The tree-like layer composed of glycans on the outer cell membrane is known as the glycocalyx. The specific biochemical composition of the glycocalyx is unique for each cell type (44). Glycans highly impact protein functions and are consequently involved in numerous biological processes including cell adhesion, signal transduction, receptor retention and endocytosis of molecules. In the context of cancer, glycans are implicated in cell invasion, regulation of vascular permeability, immune modulation, and cancer metastasis (45). Given the immunosuppressive impact of tumor metabolites, it is plausible that the interconnected metabolic and glycosylation biosynthesis pathways may contribute to these cancer phenomena. This remains, however, still a largely unexplored research area.

## 4 The tumor glycocalyx

Tumor cells are well described to have a different glycocalyx composition when compared to their healthy counterparts (46, 47). This aberrant tumor glycosylation profile has been related to the acquisition of hallmarks of cancer (48) and consequently, associated with patient outcomes. For instance, Jiang et al. (49) demonstrate that the expression of aberrant O-glycans, including the Tn antigen, in colorectal cancer is linked to tumor metastatic potential and poor prognosis. More recently, Sun et al. (50) identified a glycosylation signature for predicting the progression and immunotherapeutic response of prostate cancer, emphasizing the role of glycosylation in disease advancement and treatment outcomes.

The aberrant glycosylation profile of tumor cells is affected by various factors, including alterations in glycosyltransferase expression levels and changes in the availability of glycan substrates within the TME (51). Changes in cellular metabolic status can contribute to changes in the availability of glycan substrates, particularly through modulation of the HBP (52). Hyperglycemia, for instance, has been associated with exacerbating colon cancer malignancy through the HBP, indicating a direct relationship between glucose metabolism, HBP, and tumor progression (53, 54). Moreover, hypoxia, a common feature of the TME, has been associated with alterations in glycosylation patterns in cancer cells. Hypoxia-driven changes in glycosylation can impact cell migration and invasion, contribution to tumor aggressiveness (55).

The aberrant glycosylation profile of tumors cells impacts the interaction of tumor cells with the immune system within the TME (56–59). As an example, the aberrant O-linked glycosylation of MUC1 in carcinomas can alter the interaction of MUC1 with glycan binding receptors, consequently affecting the tumor-immune interplay (60). How alterations in tumor glycosylation affects tumor immunity, has comprehensively reviewed by others and is not the scope of this review (61–64).

Besides tumor-immune interactions, the tumor glycocalyx has also been described to be involved in the regulation of tumor cell proliferation. A cytostatic effect on tumor cells refers to the inhibition of cell proliferation without inducing cell death. This effect is crucial in cancer treatment as it aims to halt the growth and spread of tumors. Several reports propose that tumor glycoproteins may play a role in the outcome of cytostatic effects on tumor cells. The P-glycoprotein (P-gp) is a glycosylated transmembrane protein that acts as a multidrug transporter and reported to play a crucial role in multidrug resistance in cancer cells by actively removing cytostatic drugs, including chemotherapy, from tumor cells (65, 66). Whether glycosylation does impact the functionality of P-gp, has not been explored. CD44, a cell surface adhesive glycoprotein, plays a crucial role in tumorigenes. An increasing amount of literature indicates CD44, and especially the CD44v isoforms, as a marker for cancer stem cells. CD44 regulates cancer stemness, including self-renewal and metastasis (67). Hou et al. demonstrated that *N*-glycosylation of CD44 enhances its stability, consequently promoting tumor cell proliferation (68). Beyond transmembrane glycoproteins, intracellular *O*-GlcNAcylation has been linked to tumor proliferation by modulating cellular pathways (47). Inhibition of *O*-GlcNAcylation leads to accumulation of bladder cancer cells in G0/G1 phase (69). Consequently, targeting *O*-GlcNAcylation has been proposed to overcome cancer resistance to therapies including cytostatic drugs (70).

Certain subsets of T cells, particularly the regulatory T cells, as well as cancer cells are known to be able to produce transforming growth factor beta (TGFβ). TGFβ is a cytokine that plays a complex role in cancer progression. In certain contexts, TGFβ generates a population of cancer cells that reside in the G0/G1 phase with high motility and metastatic potential (71, 72). Additionally, TGFβ can induce dormancy in cancer cells, underscoring its role in maintaining quiescence in cancer cells (73). In the context of tumor dormancy, changes in TGFβ glycosylation could potentially affect its ability to induce cell cycle arrest or promote a quiescent state in cancer cells. Glycosylation alterations may influence the interaction of TGFβ with its receptors and downstream effectors, leading to differential effect on cell proliferation and dormancy (74). For instance, Sun et al. (75) demonstrates that glycosylation of TGFβ receptor II is indispensable for proper TGFβ signaling, which further promotes cell cycle arrest-like traits in breast cancer. These studies illustrate the cytostatic mechanism by which the immune system can affect cancer cells and highlight the crucial role of glycosylation alterations this process.

## 5 The T cell glycocalyx

Like the tumor cell, the T cell membrane is also covered with glycan structures. The T cell glycocalyx co-regulates key pathophysiological steps within T cell biology including T cell development, activation and proliferation (76–79). Different kinds of protein glycosylation including *O*-GlcNAcylation, fucosylation and sialylation have been described to be involved in the different stages of T cell development, from homing of T cell precursors to the thymus, to selection and maturation of single positive CD4^+^ and CD8^+^ T cells (34). The conserved Notch signaling pathway plays a major role in the initial commitment to the T cell lineage within the thymus. Early thymocyte progenitors develop in the thymus from their double negative (CD4^-^ and CD8^-^) state into T cells via the Notch pathway (80). The glycosylation profile of Notch receptors has been shown to control Notch-dependent intracellular signal transduction, stressing the relevance of glycosylation for T cell development (81). For instance, *N-*acetylglucosaminyltransferases modify Notch receptors and loss of these glycosyltransferases leads to reduced binding of Notch to Delta-like ligands, altering the frequencies of T cell subsets in the thymus (82). Mannose-restricted thymocyte glycans were found to impair key developmental checkpoints such as normal lineage choice, Treg cell generation and T cell receptor (TCR) β-selection (83). Moreover, during thymic development the reactivity of the TCR reactivity is tightly regulated and influenced by its glycosylation pattern. De-sialylation was found to enhance the sensitivity of mature T cells to low-affinity TCR ligands or self-ligands (84). Similarly, the binding ability of CD8 to MHC class I is decreased by enhanced T cell sialylation upon T cell maturation during T cell development (85). This underscores the significance of the T cell glycosylation machinery during thymic development.

The process of T cell activation typically involves three main signals. First, the TCR recognizes a specific antigen presented by antigen-presenting cells (APC) in the context of MHC molecules. Secondly, co-stimulatory molecules, such as CD28 on T cells and CD80/86 on APCs reinforces T cell activation. The third signal is obtained from cytokines released by the APC and surrounding cells that influences the differentiation, proliferation, and effector functions of the activated T cell. T cell activation leads to exceptionally high rate of growth and proliferation. Activated T cells rapidly upregulate their glucose uptake and glycolysis to fuel the energetic and biosynthetic demands for rapid clonal expansion (86). This includes generation of glycan-donor substrates required for glycan biosynthesis that is needed for proper T cell function (87). Moreover, TCR signaling induced by anti-CD3/CD28 monoclonal antibodies on T cells co-regulates mRNA expression of multiple *N*-glycan processing enzymes including MGAT5 and Golgi α-mannosidase enzymes, to promote *N*-glycan branching and formation of mature glycans (88). Involvement of glycosylation in T cell activation and sustaining their effector functions is mainly by *N*-glycosylation of the TCR, CD25 and co-stimulatory and -inhibitory receptors (34, 81, 89). Glycans can play a stabilizing role in complexes formed at the immunological synapse (34). For instance, a deficiency in β1,6 *N-*acetylglucosaminyltransferase V (MGAT5) enhances TCR clustering, resulting in a lower T cell activation (76). MGAT5 initiates GlcNAc β1,6 *N*-glycan branching (90). A deficiency in *N-*glycan branching results in lower presence of *N*-acetylglucosamine, the ligand for galectins. Galectins are known to modulate T cell proliferation and apoptosis (91) by regulating TCR clustering and recruitment to the site of antigen presentation. By removing galectin ligands, the threshold for T cell activation is lowered. The absence of MgatV has then also been associated with increased susceptibility for autoimmune disease (76). Opposingly, when inhibiting *N*-glycosylation by point mutations in *N*-glycosylation sites of CD28, CD28 showed an increased binding to CD80, leading to enhanced CD28 signaling activity (92). Collectively, *N-*glycosylation is profoundly involved in T cell activation and its impact is significantly determined by inhibiting the whole *N*-glycosylation machinery versus inhibition of *N*-glycosylation on specified T cell glycoproteins.

Besides T cell biology, the glycans on the T cell surface serve as signals for glycan binding proteins (GBPs). GBPs are widely express among a diverse set of immune cells, thereby regulating the immune response. The three main types of GBPs are galectins, Sialic acid-binding immunoglobulin-type lectins (Siglecs) and C-type lectins (93). The GBPs and their immune regulatory roles are highly diverse and complex (43, 93, 94). For example, Galectin-1 is pro-tumorigenic and proangiogenic in tumor progression. Tumor secreted Galectin-1 has immunosuppressive effects and serves as an important marker in diagnosis, prognosis, and treatment of cancer (34, 95, 96). Moreover, Galectin-1 was found to negatively influence the proliferation of CD8^+^ T cells and therefore affect antitumor immunity (97). One of the targets of Galectin-1 is the CD45 receptor on T cells. CD43 and CD45 are highly abundant glycoproteins on the T cell surface and are decorated with *O*- and *N*-glycans, regulating their function and binding. For instance, sialylation of CD45 was shown to inhibit Galectin-1-induced clustering, an initial step in Galectin-1 mediated cell death (98). This indicates that CD45 glycosylation can control T cell susceptibility to cell death (99).

Collectively, glycans serve as regulators of T cell biology, exerting significant influence on the immunological synapse, including interactions between T cells and tumor cells. Therefore, the T cell glycocalyx represents as a target to improve anti-tumor T cell immunity.

## 6 The influence of the TME on T cell functions via the T cell glycocalyx

The efficacy of T cells in inducing cancer cell arrest and elimination relies heavily on their ability to sustain functional within the TME. The TME can, however, induce T cell exhaustion and senescence, leading to altered differentiation and hypofunctional status of T cells (100, 101). Persistent antigen presentation in the TME can be associated with the induction of T cell dysfunctions, resulting in an exhausted state (101). Exhausted T cells typically exhibit heightened expression of inhibitory receptors, reduced effector cytokine production, and impaired cytolytic activity. Besides prolonged exposure to antigen, metabolites present in the TME can also influence T cell function. Elevated lactate levels, for instance, have been shown to suppress the anti-tumor activity of T cells by increasing the accumulation of H^+^ ions and maintaining a low pH environment (102, 103). In general, the functional outcome of T cells is co-determined by the activation of stimulatory and inhibitory receptors on T cells. The majority of these immune receptors are glycosylated (89, 104). The glycosylation pattern can influence the receptor’s stability, ligand binding affinity (105, 106), and recognition by therapeutic monoclonal antibodies, thus affecting their anti-tumor efficacy (107, 108). Moreover, the glycosylation machinery of a cell is dynamic and reflects the functional state of a cell. As an example, T cells present in PBMCs isolated at time of SARS-Cov-2 diagnoses (within 72h of positive PCR SARS-Cov-2 test) displayed an altered glycosylation profile when compared to healthy controls (109). A metabolic altered TME could similarly cause alterations in the T cell glycocalyx with possibly functional consequences.

Recent studies report on how *N*-glycosylation can directly interfere with T cell function within the TME. Malignant ascites fluid obtained from ovarian cancer patients, inhibited glucose uptake by CD4^+^ T cells and resulted in *N*-linked glycosylation defects. The loss of fully *N*-glycosylated proteins suppressed mitochondrial activity and IFNγ production by the CD4^+^ T cells. Restoration of *N*-linked glycosylation enhanced mitochondrial respiration again in CD4^+^ T cells exposed to malignant ovarian ascites (110). In addition to CD4^+^ T cells, Kim et al. (111) demonstrated that deficient *N*-glycosylation impairs IFNγ mediated effector function also in tumor-infiltrating CD8^+^ T cells, impacting the anti-tumor immune response. Mechanistically, tumor infiltrating and exhausted CD8^+^ T cells downregulate the oligosaccharyltransferase (OST) complex. The OST complex catalyzes the attachment of precursor *N*-glycans to nascent target proteins in the endoplasmic reticulum (ER). OST complex is therefore indispensable for the *N*-glycosylation pathway. Interestingly, restoration of the OST complex complemented *N*-glycosylation that restored the IFNγ production and alleviated CD8^+^ T cell exhaustion, consequently resulting in reduced tumor growth in preclinical models (111).

T cell proliferation and activation is dependent on the competitive binding of CTLA-4 or CD28 on the T cell to the CD80/86 ligand on an APC. When CTLA-4 binds CD80/86 instead of CD28, T cell proliferation is inhibited (112). Increased CTLA-4 glycan branching retains CTLA-4 on the cell surface, suppressing T cell activation (88). Upon activation, T cell upregulate PD-1. Upon binding of PD-1 to its ligand, PD-L1, the glycolysis metabolism is attenuated, limiting the energy supply, and impeding the differentiation into effector T cells (113). This binding also induces protumor genic rapamycin (mTOR) signaling, reactive oxygen species (ROS) production and mitochondrial respiration (114). These processes all exert negative effects on T cell activity and cytotoxicity. Immune checkpoint proteins including CTLA-4 and PD-1 are glycosylated, which can be crucial for their function (89). PD-1 contains four *N*-glycosylation sites that are critical for maintaining PD-1 membrane expression (105). Inhibition of core fucosylation enhanced the ubiquitination of PD-1, leading to PD-1 degradation by the proteasome (115). Also, glycosylation of PD-1 impacts the binding to its ligand PD-L1 (105). Similarly, recognition of PD-1 by the anti-PD-1 blocking antibody Camrelizumab used for the treatment of relapsed or refractory classical Hodgkin lymphoma (116) is affected by PD-1 glycosylation (108). Whether changes in glycosylation of immune checkpoints occur within the tumor microenvironment has, however, not extensively been researched. First studies show that elevated glycosylation on tumor cells results in overexpression of PD-L1, therefore increasing its immunosuppressive activity (106, 117). However, whether the TME can affect PD-1 glycosylation remains largely unstudied.

Glyco-metabolism changes could possibly also indirectly impact T cell function by affecting glycans on extracellular matrix (ECM) components in the TME and hence binding of secreted factors such as immunomodulatory cytokines and chemokines. IL-2, TGFβ and IFNγ are for instance known to bind the ECM glycosaminoglycans such as heparin sulfate and this binding modulates the biological activity of these cytokines (118–120).

**Figure 2:**
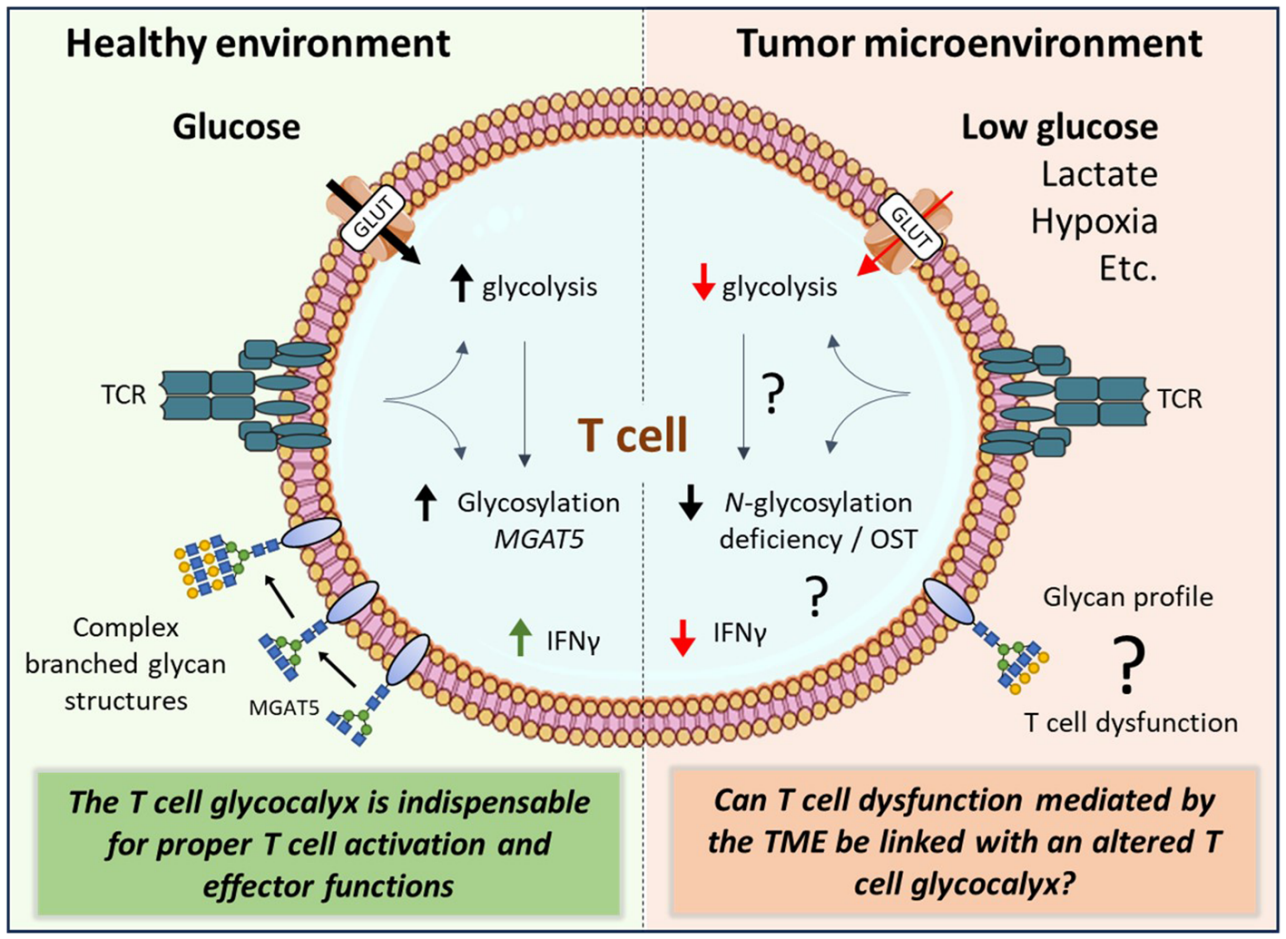
The complex interplay between the tumor microenvironment and the T cell glycocalyx. Activated T cells rapidly increase their glucose uptake to fuel their energetic demands. The tumor microenvironment (TME), however, is deprived from nutrients including glucose. The TME can induce T cell exhaustion and senescence. Although glycolysis and glycan biosynthesis pathways are highly interconnected, it remains unknown how TME factors affect T cell effector functions through changes in glycan biosynthesis of T cells. glucose transporter (GLUT), β1,6 N-acetylglucosaminyltransferase V (MGAT5), oligosaccharyltransferase (OST), interferon gamma (IFNγ), T cell receptor (TCR).

In summary, the T cell glycocalyx is indispensable for proper T cell activation and effector functions (**Figure 2**). Glycan biosynthesis requires glucose molecules, yet the levels of glucose in the TME are significantly lower compared to those in non-malignant tissue. To gain a deeper understanding of how the TME impacts T cell biology, future research should encompass the influence of the TME on the T cell glycocalyx, potentially leading to T cell dysfunction (see ‘?’, **Figure 2**).

## 7 Conclusion & Future perspectives

The onco-immunology field has witnessed significant advancements, in part driven by immunotherapies such as immune checkpoint and CAR T cell therapy. Although immunotherapies have demonstrated clinical success, T cell infiltration alone does not determine efficacy. Tumor cells exhibit altered metabolism, notably the Warburg effect, impacting glucose consumption and lactate production, thereby fostering a metabolically restricted TME. This influences the competition for glucose and other nutrients between tumor and immune cells. Most of the proteins expressed on T cells require glycosylation to function properly. The competition for glucose might have a direct effect on the glycosylation profile of T cell proteins and consequently affecting T cell effector functions. The key question that now arises is: How exactly does the TME influence the T cell glycocalyx and can this be linked with T cell functioning? There is evidence that T cells are exhausted inside the TME because of glucose-restriction, hypoxia, and elevated lactate levels, but can this be linked with an altered glycosylation machinery within the T cell?

Collectively, understanding the intricate connection between tumor glucose metabolism, the T cell glycocalyx and T cell biology will provide new insights for advancing immune checkpoint and adoptive (CAR) T cell immunotherapy. Targeting these interconnected pathways may provide new avenues for enhancing therapeutic efficacy and overcoming challenges in the rapidly evolving landscape of onco-immunology.

## Author contributions

FS: Writing – original draft, Writing – review & editing. KW: Writing – original draft. GA: Conceptualization, Funding acquisition, Supervision, Writing – review & editing. LC: Conceptualization, Funding acquisition, Supervision, Writing – original draft, Writing – review & editing.
